# Introduction to *RSC Chemical Biology*

**DOI:** 10.1039/d0cb90001j

**Published:** 2020-04-16

## Abstract

Welcome to the inaugural issue of *RSC Chemical Biology*, a Gold open-access journal dedicated to publishing breakthrough research at the interface of chemistry and biology.
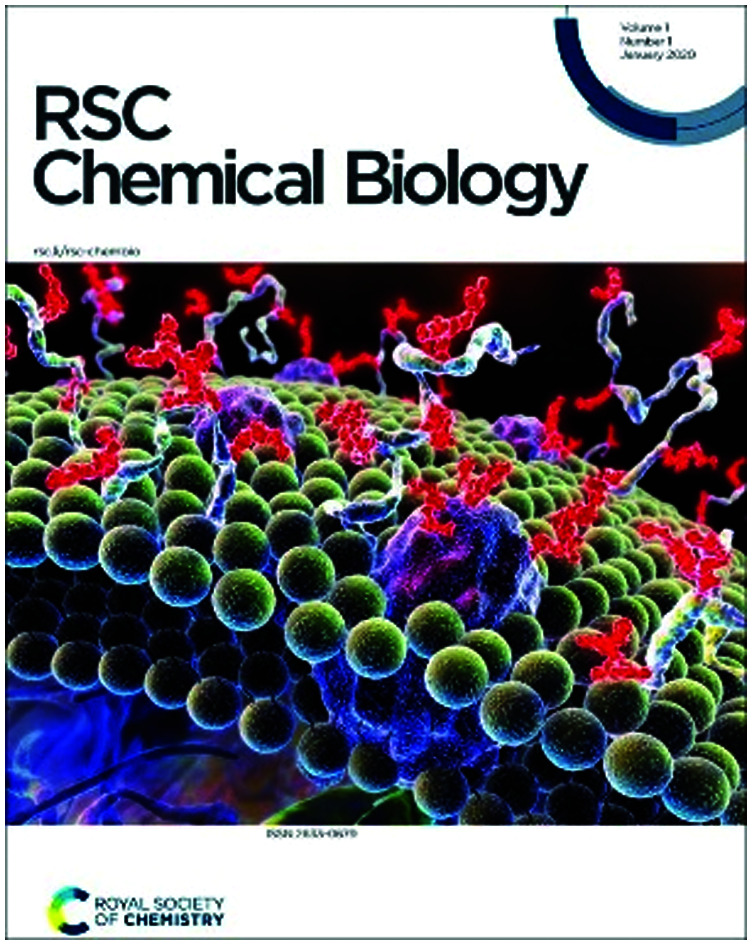

## Introduction

Welcome to the inaugural issue of *RSC Chemical Biology*, a Gold open-access journal dedicated to publishing breakthrough research at the interface of chemistry and biology. We are delighted to launch this journal to broaden the Royal Society of Chemistry portfolio and create a publishing platform for research at the frontier of chemistry and biology. We will support our community by providing them with a dedicated venue open for conversation, exchange of ideas, and collaboration, and until mid-2022, the journal is completely free to publish in.
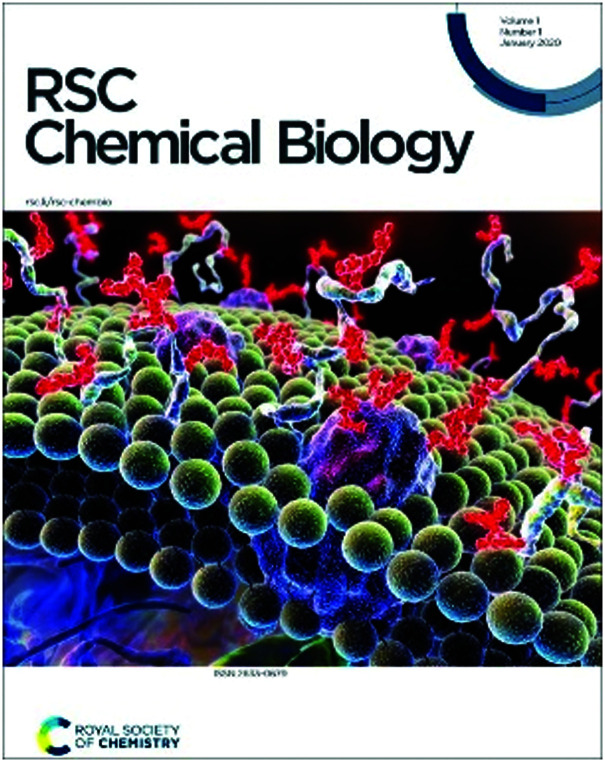


We present a truly modern product that aims to become a home for high-**quality** research where our editorial and data policies safeguard the **reproducibility** of published papers. We collaborate with Kudos which helps our authors to optimize the website traffic and in result increase the **discoverability** of their papers. A Gold open-access model coupled with extensive content promotion gives our authors enhanced **visibility**. In addition to the above, we are offering a pioneering RSC transparent peer-review process to show our commitment to **transparency** and open science.

## Journal scope

*RSC Chemical Biology* welcomes contributions that integrate concepts and approaches of chemistry, biology, and related disciplines that examine and engineer complex biological systems. We particularly invite papers that propose innovative solutions to existing challenges and help to drive research forward. We aim to position this new journal as an authoritative platform to exchange knowledge and ideas and foster collaboration between chemical and biological communities, as well as academia and industry, and thus we welcome the submission of Opinions and Comments. All published articles must be of significant general interest to the broad chemical biology community.

## Editorial Board

The Editorial Board of *RSC Chemical Biology*, led by Hiroaki Suga (University of Tokyo), comprises leading scientists in the field dedicated to and expert in discerning state-of-the-art developments and achievements in the chemical biology discipline.
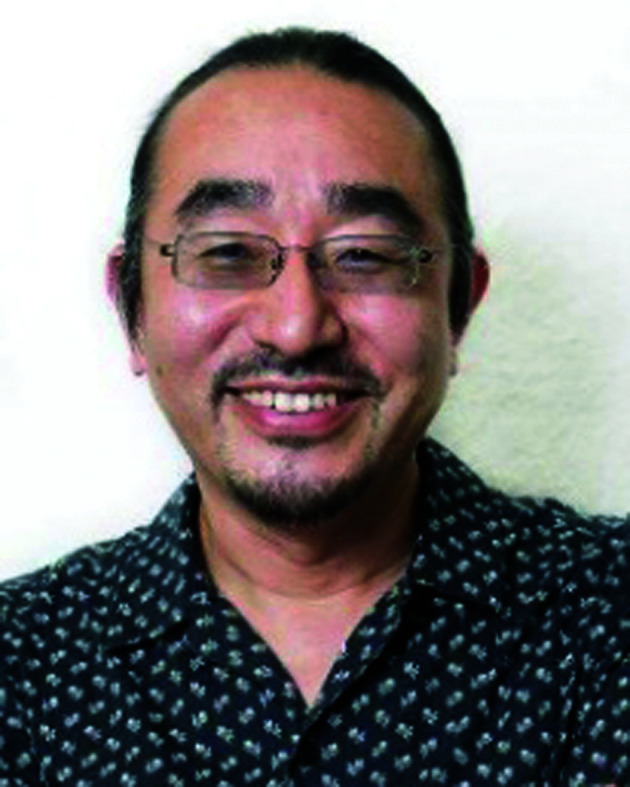


Hiroaki Suga, Editorial Board Chair, *RSC Chemical Biology*

**Hiroaki Suga** leads the journal in the role of Editorial Board Chair. He is a Professor of the Department of Chemistry, Graduate School of Science at the University of Tokyo. With broad research interests in the fields of bioorganic chemistry, chemical biology and biotechnology related to RNA, translation, and peptides and with a wide international collaboration network, he is perfectly placed to recognize the needs of the community and shape the journal accordingly.

“I am very excited to be involved in this new Royal Society of Chemistry initiative. The notion of chemical biology has been aggressively growing in the fields of chemistry and biology, and I believe that this trend will continue expanding into pharmaceutical science and translational research and further into materials and information technologies. *RSC Chemical Biology* will cover all these interdisciplinary areas, and most importantly the journal will keep the high quality of publications along with rapid and fair peer-review.”

## Transparent peer-review

The Royal Society of Chemistry journals are recognized by the community for their quality review process. The same holds true for *RSC Chemical Biology.* We strive to publish only thorough and credible research and thus engage outstanding researchers to handle our peer-review and ensure the process is **fair** and **rigorous**. At the same time, we aim to serve authors and meet their needs and expectations, making sure that our peer-review is **timely** and **constructive**. Now we have decided to take a step forward and innovate the process by making it even more open and **transparent**.

*RSC Chemical Biology* is the first Royal Society of Chemistry journal to offer our authors the option of Transparent Peer-Review. Authors are given a choice as to whether they want to publish the peer-review history for their paper. If they decide to do so, the editor's decision letter, reviewers’ comments and authors’ response for all versions of the manuscript are published alongside the article under an Open Access Creative Commons license (CC-BY). In this way, we bring peer-review to light and acknowledge our community of reviewers for the work they put into shaping our publication landscape. You can read through the first openly published peer-review here.

## What's next?

In the first issue, we cover trending topics in Chemical Biology and we hope that you enjoy reading the first papers. We will strive to retain this quality level in the future and invite you to help us build this journal by joining our upcoming initiatives.

Follow us on Twitter or Facebook, or sign up to our newsletter to keep up to date with the latest news and some of our most recently published content.

Finally, we are committed to developing and taking *RSC Chemical Biology* forward so that the journal fully meets the needs of our authors and readers. We always welcome comments, suggestions, and feedback, so please do contact us at chembio-rsc@rsc.org with your views and feedback.

 

Professor Hiroaki Suga, Editorial Board Chair

Dr Anna Rulka, Executive Editor

Kathryn L. Gempf, Deputy Editor

## Supplementary Material

